# The isoprenyl chain length of coenzyme Q mediates the nutritional resistance of fungi to amoeba predation

**DOI:** 10.1128/mbio.00342-24

**Published:** 2024-05-15

**Authors:** Nauman Saeed, Vito Valiante, Johann E. Kufs, Falk Hillmann

**Affiliations:** 1 Evolution of Microbial Interactions, Leibniz Institute for Natural Product Research and Infection Biology (HKI), Jena, Germany; 2 Institute of Microbiology, Faculty of Biological Sciences, Friedrich Schiller University Jena, Jena, Germany; 3 Biochemistry/Biotechnology, Faculty of Engineering, Wismar University of Applied Sciences Technology, Business and Design, Wismar, Germany; 4 Biobricks of Microbial Natural Product Syntheses, Leibniz Institute for Natural Product Research and Infection Biology (HKI), Jena, Germany; 5 Genome Engineering and Editing, Faculty of Technology, Bielefeld University, Bielefeld, Germany; Universidade de Sao Paulo, Ribeirao Preto, Sao Paulo, Brazil

**Keywords:** yeast, amoeba, *Saccharomyces*, *Candida*, predation, ubiquinone, coenzyme Q

## Abstract

Amoebae are environmental predators feeding on bacteria, fungi, and other eukaryotic microbes. Predatory interactions alter microbial communities and impose selective pressure toward phagocytic resistance or escape which may, in turn, foster virulence attributes. The ubiquitous fungivorous amoeba *Protostelium aurantium* has a wide prey spectrum in the fungal kingdom but discriminates against members of the *Saccharomyces* clade, such as *Saccharomyces cerevisiae* and *Candida glabrata*. Here, we show that this prey discrimination among fungi is solely based on the presence of ubiquinone as an essential cofactor for the predator. While the amoeba readily fed on fungi with CoQ presenting longer isoprenyl side chain variants CoQ8-10, such as those from the *Candida* clade, it failed to proliferate on those with shorter CoQ variants, specifically from the *Saccharomyces* clade (CoQ6). Supplementing non-edible yeast with CoQ9 or CoQ10 rescued the growth *of P. aurantium*, highlighting the importance of a long isoprenyl side chain. Heterologous biosynthesis of CoQ9 in *S. cerevisiae* by introducing genes responsible for CoQ9 production from the evolutionary more basic *Yarrowia lipolytica* complemented the function of the native CoQ6. The results suggest that the use of CoQ6 among members of the *Saccharomyces* clade might have originated as a predatory escape strategy in fungal lineages and could be retained in organisms that were able to thrive by fermentation.

**IMPORTANCE:**

Ubiquinones (CoQ) are universal electron carriers in the respiratory chain of all aerobic bacteria and eukaryotes. Usually 8-10 isoprenyl units ensure their localization within the lipid bilayer. Members of the *Saccharomyces* clade among fungi are unique in using only 6. The reason for this is unclear. Here we provide evidence that the use of CoQ6 efficiently protects these fungi from predation by the ubiquitous fungivorous amoeba *Protostelium aurantium* which lacks its own biosynthetic pathway for this vitamin. The amoebae were starving on a diet of CoQ6 yeasts which could be complemented by either the addition of longer CoQs or the genetic engineering of a CoQ9 biosynthetic pathway.

## INTRODUCTION

Fungi are ubiquitous organisms that inhabit various environments, including terrestrial, aquatic, and even extremes such as deserts and arctic glaciers. Depending on the ecological niche, fungi interact with other microorganisms, of which mutual associations between fungi and algae or cyanobacteria (lichens) or between fungi and plant roots (mycorrhiza) are among the best-characterized examples (1). Predatory interactions between fungi and other microorganisms are also widespread, with fungi acting either as predators or prey. Fungi may feed on nematodes and other invertebrates or may, in turn, become prey for fungivore insects, nematodes, or amoebae (1).

Fungivory is taxonomically widespread among the amoebozoa kingdom and was first described for *Acanthamoeba castellanii,* which was isolated with its likely food source, the basidiomycetous yeast *Cryptococcus neoformans* (2). Further examples of amoeba mycophagy include Vampyrellid amoebae perforating and feeding on fungal spores (3). The unicellular eukaryote *Protostelium aurantium* is a member of the amoebozoa, and so far, fungi are its only accepted food source. Cells of *P. aurantium* feed on their fungal prey using two distinct mechanisms: either single-cell yeasts are engulfed by phagocytosis or hyphae of the filamentous fungi are exploited by protoplast feeding with protrusions of the amoebae piercing the fungal cell wall and invading the fungal cytoplasm (4).

As a widespread inhabitant of deciduous trees, a habitat shared by many fungal species, fungal predation by members of the Protostelia could have shaped the microverse and may have fueled the ramification of the fungal evolutionary tree. This idea is further supported by the wide taxonomic range of fungi that support the growth of these amoebae. Several representatives of the ascomycetes and basidiomycetes were previously found to be eligible food sources, for example, nearly all tested members of the highly diverse *Candida* clade were readily recognized and consumed. Even the commensal-borne human pathogen *Candida albicans* could, in principle, serve as a food source, although its mannoprotein-rich cell surface provided some protection from initial recognition. Surprisingly, no member of the closely related *Saccharomyces* clade, including bakers’ yeast *Saccharomyces cerevisiae* or its pathogenic relative *Candida glabrata,* could support the growth of *P. aurantium*, although the cells were readily taken up (4). As the amoebae could grow well on a mixed diet of cells from the *Candida* and *Saccharomyces* clade, we concluded that the *Saccharomyces* yeasts alone lacked a crucial nutritional factor provided by other yeast species. Indeed, one of the rare differences between *Candida* and *Saccharomyces* cells is the use of different ubiquinone cofactors.

**Fig 1:**
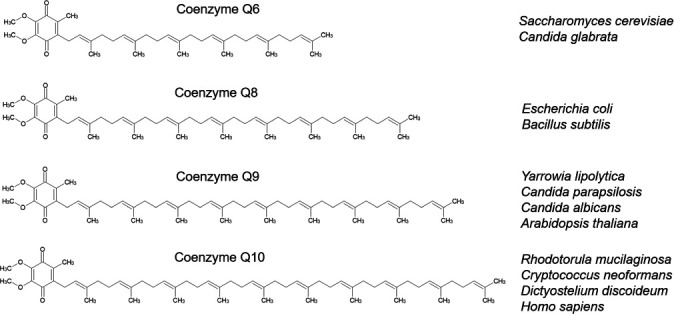
Distribution of ubiquinone (CoQs) variants among different organisms. The figure was made using ChemSketch.

Ubiquinones (CoQs) are universal electron carriers in the respiratory chain of all aerobic bacteria and eukaryotes. While nearly all fungi use CoQs with chain lengths between 7 and 10 isoprenyl units, members of the *Saccharomyces* clade, including *S. cerevisiae* and *C. glabrata* are rather unique in using only 6 (5–7) (Fig. 1). The reason for this difference is unclear, although it has recently been shown that longer CoQ side chains can contribute to higher farnesol resistance in yeasts (8). Here, we show that fungi-supplied CoQ is an essential dietary supplement for the amoebae and that the isoprenyl-chain length of the prey fungus alone is crucial to escape the predator. These results presented here suggest that the unusual CoQ chain lengths among the *Saccharomyces* clade have evolved to escape predation.

## MATERIALS AND METHODS

### Strains and growth conditions

*P. aurantium* var. *fungivorum* (9) was cultured in 10 mM potassium phosphate buffer (KK2, 0.003M K_2_HPO_4_, 0.007 KH_2_PO_4_, pH 6.4) containing 5 µg mL^−1^ kanamycin (Merck) with *Rhodotorula mucilaginosa* as a sole food source at 22°C. *E. coli* transformants were grown in LB media containing 100 µg mL^−1^ ampicillin (Merck). *R. mucilaginosa*, *Y. lipolytica*, *Candida parapsilosis,* and *S. cerevisiae* wild-type strains were grown in YPD medium at 30°C, whereas *S. cerevisiae* mutant strains (COQ1^SC^, COQ1^YL^) were cultured in synthetic complete (Kaiser SC Double Drop-Out, -His, -Leu, Histidine, and Leucine) media containing 400 µg mL^−1^ geneticin G418 (Invivogen) with 2% glucose or 4% glycerol at 30°C, otherwise mentioned. 1.5% (wt/vol) agar was added for the growth on solid media. Detailed genotypes of all the strains used in this study are provided in Table S1.

### Plaque assays

For the rapid screening of food sources *R. mucilaginosa*, *Y. lipolytica,* and *S. cerevisiae* (WT, COQ1^SC^, or COQ1^YL^), cells were streaked on KK2 agar plates. *P. aurantium* trophozoites from a pre-culture agar block were then placed upside down in the middle of the plate. Furthermore, to quantify the growth efficiency of *P. aurantium*, yeast strains (Table S1) were grown in YPD until an OD 40, harvested by centrifugation at 3,000 × *g* for 5 min at 4°C, and washed twice with ddH_2_O. The cells were then spread on 10 mM KK2 agar plates. Moreover, *S. cerevisiae* WT cells only and premixed with 10 µM CoQ6, CoQ9, or CoQ10 (Merck) and *S. cerevisiae* (WT, COQ1^SC^, or COQ1^YL^) cells mixed with *R. mucilaginosa* at 1:1, 1:10, or 10:1 ratio were also spread on 10 mM KK2 agar plates. Afterward, 10^4^ trophozoites of *P. aurantium* are added to the yeast lawn’s center. The plaque diameter formed by *P. aurantium* was measured every 24 h for 7 days otherwise mentioned.

### Genomic DNA extraction

*S. cerevisiae* and *Y. lipolytica* cells were harvested and washed twice after overnight incubation in YPD media at 30°C and 180 rpm. Approximately 100–200 mg of cells (fresh weight) was transferred to an Eppendorf tube containing 0.5 mm glass beads, resuspended in 600 µL of lysis buffer (Tris 0.05 M, pH 7; SDS 3%; EDTA 0.05 M), and vortexed vigorously. The lysate was then incubated at 65°C for 15 min and placed on ice for 5 min. Next, 300 µL of 5 M KAc was added, vortexed for 10 s, and centrifuged for 15 min at 13,000 rpm. The supernatant was transferred to a new Eppendorf tube containing 800 µL ice-cold isopropanol and incubated for >1 h at −20°C. The DNA was precipitated by centrifugation for 20 min at 4°C and 13,000 rpm and washed twice with 500 µL of 70% (vol/vol) ethanol. The pellet was then dried at 65°C and resuspended in 100 µL of sterile distilled water. The concentration of DNA was measured with a spectrophotometer (NanoDrop Technologies Inc., USA) at 260 nm.

### Construction of plasmids

To express *Y. lipolytica* (Gene ID: 2909963) and *S. cerevisiae COQ1* gene (Gene ID: 852288) in *S. cerevisiae* under the native promoter, pJet1.2_Sc-coq1 and pJet1.2_Yl-coq1 plasmids were generated using pJet1.2_rec as a template plasmid. We aligned *S. cerevisiae* Coq1 (XP_009957.1) with *Y. lipolytica* Coq1 (XP_501989.1) using the BLASTp program at default settings to replace the *Y. lipolytica* signal peptide with the *S. cerevisiae*. The mitochondrial import signal (MIS) of the *S. cerevisiae* Coq1 covers the first 53 amino acids (10). Based on the alignment, we replaced the N-terminal 60 amino acids of *Y. lipolytica* Coq1 with the sequence covering the first 56 amino acids of *S. cerevisiae* Coq1 (hexaprenyl pyrophosphate synthase). Subsequently, we PCR amplified the 1,119 bp fragment of Yl-*coq1*, excluding the first 180 nucleotides, using overhang primers Yl_coq1_1 and Yl_coq1_2. We cloned this fragment next to the *S. cerevisiae* MIS in the pJet1.2 vector, resulting in the pJet1.2_Yl-coq1 plasmid. Likewise, we PCR amplified the 1254 bp fragment of Sc-*COQ1*, excluding the first 168 nucleotides, using overhang primers Sc_coq1_1 and Sc_coq1_2, and cloned it next to the *S. cerevisiae* MIS in the pJet1.2_rec vector, creating the pJet1.2_Sc-coq1 plasmid. All the primers used in this study are listed in Table S2.

After generating these plasmids, we introduced them into competent TOP10 *E. coli* cells, and colonies were selectively grown on LB agar plates containing 100 µg mL^−1^ of ampicillin.

### Cloning in *Saccharomyces cerevisiae*

For homologous recombination in *S. cerevisiae*, primer pair pJet1.2_rec_fwd and pJet1.2_rec_rev was used to amplify 4,822 bp and 4,687 bp fragments from pJet1.2_Sc-coq1 and pJet1.2_Yl-coq1 plasmids, respectively. The PCR-amplified fragments were then transformed in the *S. cerevisiae* BY4741 strain to generate Sc*coq1*::Sc*coq1* (COQ1^SC^) and Sc*coq1*::Yl*coq1* (COQ1^YL^) mutant strains, respectively. These mutants were selected on YPD agar plates containing 400 µg mL^−1^ geneticin G418 antibiotic, and the correct transformants were then confirmed by PCR amplification using Sc_5′ Check and Sc_3′ Check primers. All primers are listed in Table S2.

### Genes and proteins involved in CoQ biosynthesis

The sequences of genes and proteins involved in CoQ biosynthesis in *Dictyostelium discoideum* were retrieved from the National Center for Biotechnology Information (NCBI) protein database and used to query the NCBI protein database using the protein Basic Local Alignment Search Tool (BLASTp) by selecting organism *P. aurantium var. fungivorum* and other parameters as default.

### Coenzyme Q extraction

The CoQ was extracted according to Pierrel and colleagues with few modifications (11). Yeast cells were freshly grown in a synthetic complete media containing 4% glycerol until they reached the stationary phase. They were washed twice with sterile distilled water at 3,000 × g for 5 min and resuspended in 5 mL water. Then, the cells were lysed with 0.5 mm glass beads (10 mL) using a mixer mill (MM400, Retsch) for 6 min at 30 Hz, and subsequently, 18 mL of methanol and 12 mL of petroleum ether were added. Samples were shaken again with the mixer mill at 10 Hz for 3 min and centrifuged at 5,000 × *g* for 5 min. The transparent petroleum ether phase was transferred into the round bottom flask. CoQ was re-extracted with 12 mL of petroleum ether twice. The solvent was then evaporated using a rotary evaporator, and the pellet was resolved in 1 mL of chloroform/methanol (2:1 [vol/vol]). Afterward, the extracted coenzyme Q samples were concentrated by drying under pressurized air and re-dissolved in 200 µL of chloroform/methanol (2:1 [vol/vol]).

### TLC and HPLC

The concentrated coenzyme Q extracts were analyzed by normal phase thin-layer chromatography (TLC) and high-performance liquid chromatography (HPLC), with slight modifications as previously reported (12). Normal-phase TLC was conducted on a Kieselgel 60 F254 plate (Merck Millipore) and was developed with benzene for separation for 30 min. The plate was viewed under UV illumination, the CoQs band was collected, and the sample was extracted with ≥99.8% ethanol. Purified CoQs were subjected to high-performance liquid chromatography on a Shimadzu HPLC LC-20AD instrument equipped with a reverse-phase Nucleodur C18 gravity column (4.6 × 150 mm, 5 µm, 110 Å, Macherey-Nagel). Isocratic separation was performed with methanol containing 35% ethanol and a flow rate of 1 mL/min. CoQs were detected at 275 nm using a PDA detector (Shimadzu).

### Statistical analysis

GraphPad was used for all statistical analysis, and all error bars represent ± standard deviation from at least three biological replicates. Student’s *t*-test with the following significance *P < 0.1; **P < 0.05; ***P < 0.01 was used.

## RESULTS

### *P. aurantium* lacks a functional coenzyme Q biosynthesis pathway

**Fig 2:**
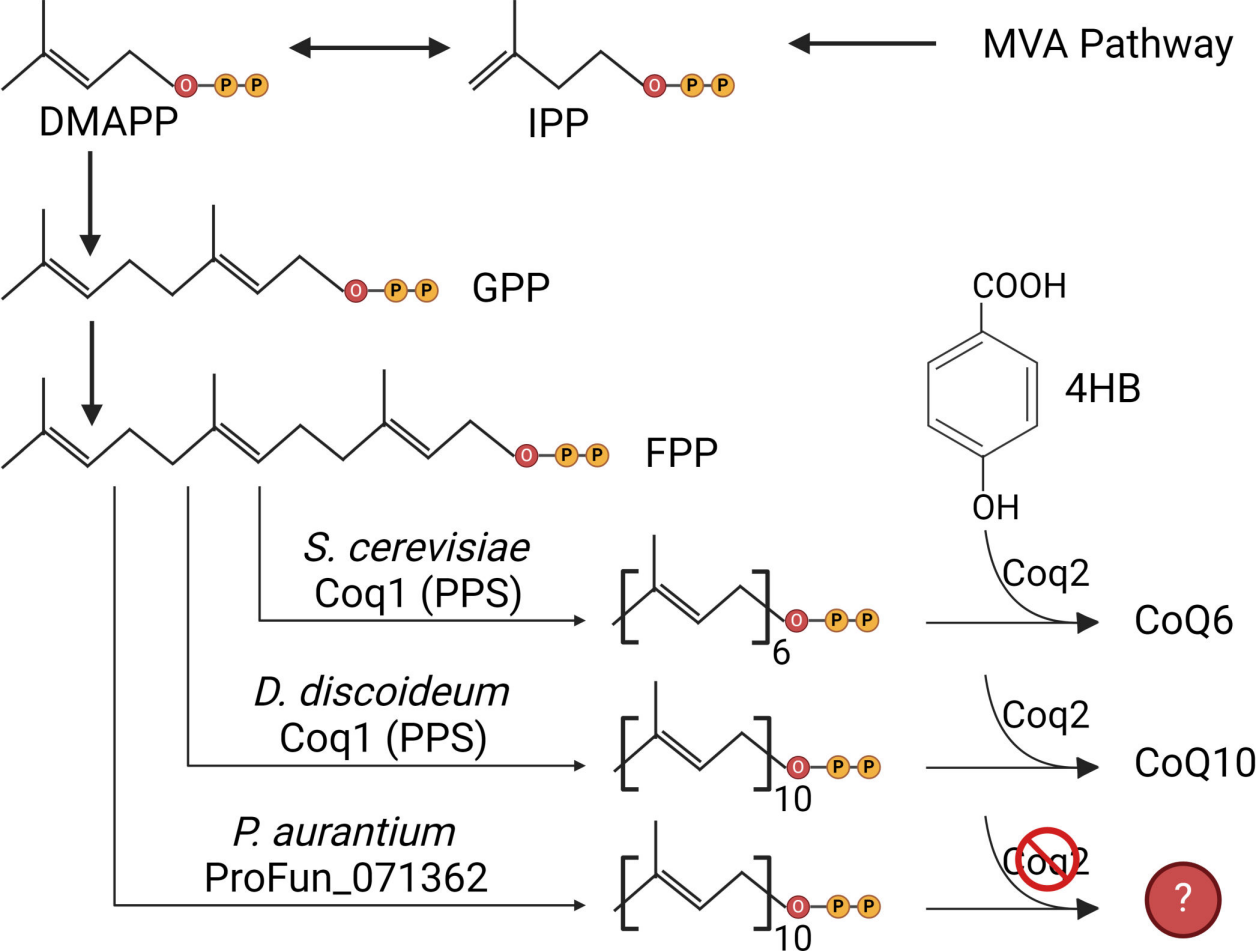
Ubiquinone biosynthesis pathway in eukaryotes. MVA, mevalonate; IPP, isopentenyl pyrophosphate; DMAPP, dimethylallyl diphosphate; GPP, geranyl diphosphate; FPP, farnesyl diphosphate; PPS, polyprenyl synthase; 4HB, 4-hydroxybenzoic acid. The figure was made using BioRender.

*P. aurantium* strongly discriminated against fungal cells of the *Saccharomyces* clade as food sources when compared to those of the *Candida* clade. One of the few fundamental molecular differences between the two clades is the CoQ-variants (7). To determine whether CoQ could be an essential factor that *P. aurantium* obtains solely *via* its fungal prey, we investigated the genomic basis of the CoQ biosynthetic pathway in *P. aurantium*. In *S. cerevisiae,* CoQ biosynthesis is fueled by the mevalonate pathway, and 13 genes (*COQ1–COQ11*, *YAH1*, and *ARH1*) are involved in the biosynthesis and efficient functioning of CoQ (13, 14). While only nine genes (*COQ1–COQ9*) are directly implicated in CoQ6 biosynthesis in *S. cerevisiae* (13), we sought *D. discoideum* orthologs of *coq1-coq9* in *P. aurantium var. fungivorum*. To our surprise, potential orthologs of only four *D*. *discoideum coq* genes (*coq1*, *coq3*, *coq5*, and *coq6*) were identified in the genome of *P. aurantium* (Table 1). These results suggest that *P. aurantium* is unlikely to operate a functional coenzyme Q biosynthesis pathway (Fig. 2) and instead depends on its prey to acquire CoQ for respiration.

**TABLE 1:** Genes associated with the biosynthesis of ubiquinone in *D. discoideum* and their closest orthologues in *P. aurantium*

No.	Dicty ID	UniProt ID	Gene	Function	Putative ortholog in *P. aurantium*	Identity %	Annotation
1	DDB_G0280293	Q54VJ9	*coq1*	Decaprenyl-diphosphate synthase	PROFUN_07136	25.80	Polyprenyl synthetase
2	DDB_G0281241	Q54U71	*coq2*	*p*-hydroxybenzoate (PHB) polyprenyltransferase	None found		
3	DDB_G0279037	Q54XD0	*coq3*	Polyprenyldihydroxybenzoate methyltransferase	PROFUN_09622	28.12	Putative 3-demethylubiquinone-9 3-methyltransferase
					PROFUN_02854	50	Protein arginine N-methyltransferase 1
4	DDB_G0292620	Q54CZ2	*coq4*	Unknown	None found		
5	DDB_G0280237	Q54VN2	*coq5*	2-Methoxy-6-polyprenyl-1,4- benzoquinol methylase	PROFUN_11016	21.10	S-adenosyl-methionine-sterol-C-methyltransferase Hypothetical protein
					PROFUN_12575	24.06	Methyltranferase type 11
					PROFUN_12350	31.94	Hypothetical protein
6	DDB_G0291440	Q54EN1	*coq6*	Ubiquinone biosynthesis monooxygenase	PROFUN_09882	21.79	2-Polyprenyl-6-methoxyphenol hydroxylase-like oxidoreductase
					PROFUN_13178	21.59	Hypothetical protein
					PROFUN_13834	33.78	Hypothetical protein
					PROFUN_00446	22.52	2,4-Dichlorophenol 6-monooxygenase
					PROFUN_15084	34.48	2-Polyprenyl-6-methoxyphenol hydroxylase-like oxidoreductase
7	DDB_G0280475	Q54VB3	*coq7*	5-Demethoxyubiquinone hydroxylase	None found		
8	DDB_G0288749	Q54IH6	*abkA*	Protein kinase	None found		
9	DDB_G0274457	Q86HS0	*coq9*	Unknown	None found		

### *P. aurantium* requires CoQ with a long isoprenyl side chain for its growth

**Fig 3:**
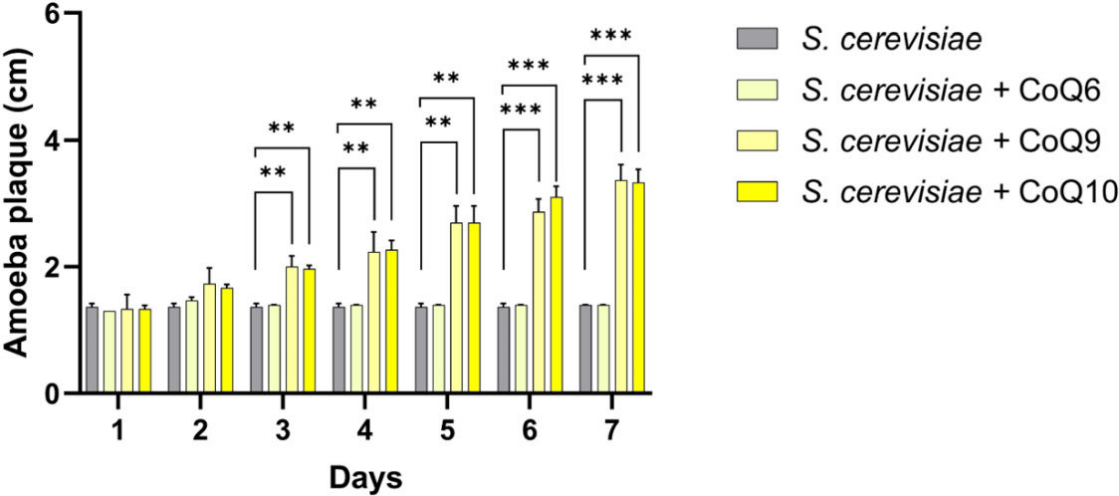
Growth of *P. aurantium* on *S. cerevisiae* alone or supplemented with exogeneous CoQs. Amoeba plaques formed by the feeding of *S. cerevisiae* alone or with 10 µM CoQs (Coq6, Coq9, or CoQ10) on potassium phosphate agar plates were measured daily for 7 days and plotted on the graph. The data are represented as the mean values with error bars from three biological replicates. Significance levels are denoted with asterisks, where **P < 0.05; ***P < 0.01, based on *t*-test analysis.

Although *P. aurantium* possesses an ortholog of *D. discoideum* decaprenyl pyrophosphate synthase (PROFUN_07136), the fact that it lacks orthologs for *coq2*, *coq7*, *coq8*, and *coq9,* essential genes for coenzyme Q biosynthesis, made it seem likely that *P. aurantium* would still depend on its prey for CoQs for respiration. As the amoeba was unable to maintain its growth on fungal species with CoQ6, such as *S. cerevisiae*, we hypothesized that *S. cerevisiae* exogenously supplemented with CoQ9 or CoQ10 could lead to sustained growth on an otherwise inedible food source. Indeed, after 7 days of predation, plaque diameters on *S. cerevisiae* with supplemented CoQ9 or CoQ10 increased over time, while no growth was observed with *S. cerevisiae* alone or with CoQ6 (Fig. 3). These results suggest that exogenous supplementation of CoQ with a long isoprenyl side chain rescues the growth of *P. aurantium* on non-food source members of the *Saccharomyces* clade and that *P. aurantium* depends on external provision of this cofactor.

### Heterologous biosynthesis of CoQ9 in *S. cerevisiae*

**Fig 4:**
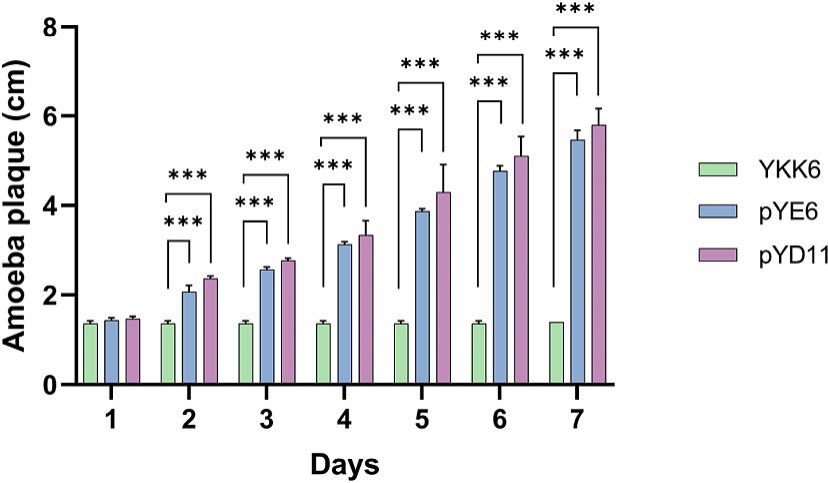
Growth of *P. aurantium* on YKK6, pYE6 and pYD11 strains. Amoeba plaques formed by the feeding of *S. cerevisiae* YKK6, pYE6, or pYD11 strains on potassium phosphate agar plates were measured daily for 7 days and plotted on the graph. The data are represented as the mean values with error bars from three biological replicates. Significance levels are denoted with asterisks, where ***P < 0.01, based on *t*-test analysis.

Furthermore, we wanted to determine whether the heterologous expression of genes involved in the production of coenzyme Q with a long isoprenyl side chain in *S. cerevisiae* could complement the function of *COQ1* in *S. cerevisiae. S. cerevisiae* strains synthesizing long-chain CoQ were previously constructed to study the biosynthetic pathway of CoQs (15). When using these strains harboring plasmids to produce CoQ8 (pYE6) and CoQ10 (pYD11), we observed sustained feeding of *P. aurantium* on these cells, compared to the YKK6 strain (Δ*coq1*) (Fig. 4). We further constructed yeast strains in which we replaced the *S. cerevisiae COQ1* gene directly in the *COQ1* locus. *Y. lipolytica* was chosen as a genetic donor because this yeast was known to synthesize CoQ9 and likely harbors a phylogenetically more ancient version of the fungal COQ1 gene. A locus-specific exchange with the *S. cerevisiae’s COQ1* was also carried out as a control. To deliver the *Yl*-polyprenyl synthase into mitochondria, the gene was fused with a sequence encoding the 56 amino acids containing the mitochondrial import signal (MIS) of Sc-*COQ1*. Integration to the correct genetic locus was verified by PCR (Fig. S1).

**Fig 5:**
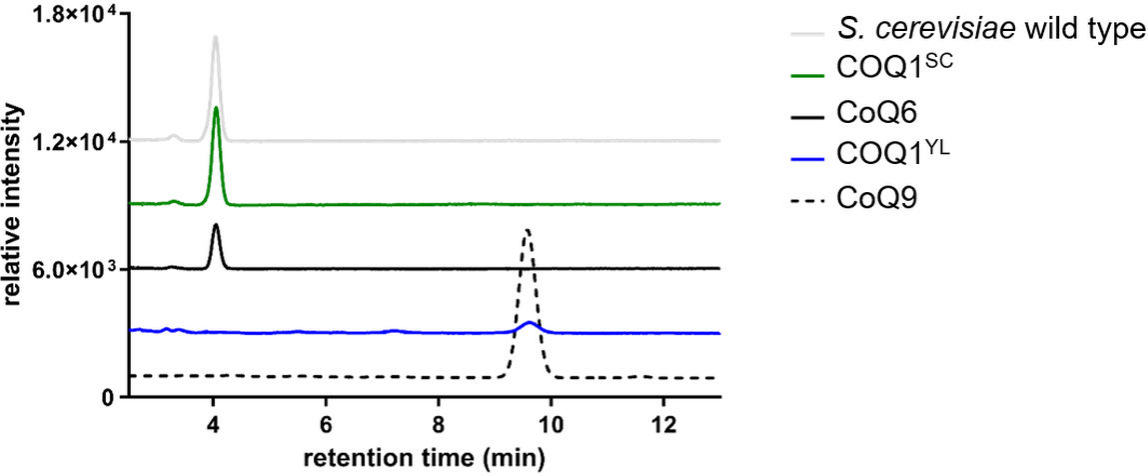
HPLC analysis of COQs produced by *S. cerevisiae* strains, wild type, COQ1^SC^, or COQ1^YL^. HPLC analysis confirmed the loss of the CoQ6 peak (4 min) and the presence of a CoQ9 peak (9.6 min) for the COQ1^YL^ strain. The negative control COQ1^SC^ in which the gene COQ1 was exchanged with the endogenous gene still shows the presence of CoQ6.

Following the successful generation of mutants, we extracted coenzyme Q from COQ1^SC^ and COQ1^YL^ strains using the petroleum ether-methanol extraction method to analyze the functional capability of Sc-*COQ1* and Yl-*coq1* to produce coenzyme Q, respectively. Following extraction and thin layer chromatography, bands corresponding to possible coenzyme Q6 and coenzyme Q9 were visualized under UV illumination in the extracts from COQ1^SC^ and COQ1^YL^ strains, respectively (Fig. S2). The bands were collected and extracted with ethanol for HPLC analysis, confirming their identity as CoQ6 and CoQ9, respectively (Fig. 5).

**Fig 6:**
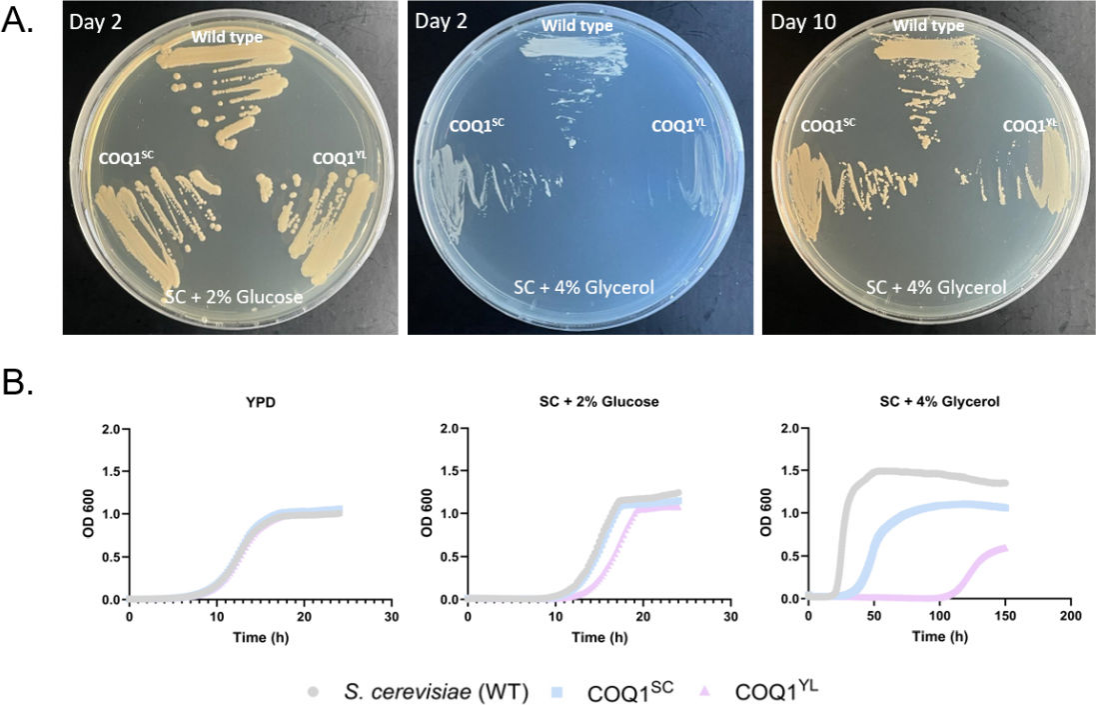
Growth of *S. cerevisiae* wild type (WT), COQ1^SC^, and COQ1^YL^ in synthetic complete media with either glucose or glycerol as carbon sources. (**A**) Cells were streaked on a solid medium, and pictures were taken after 2 and 10 days. (**B**) Growth in a liquid medium was determined by following the OD_600_ over a time course of 24 h (YPD), 24 h (SC + 2% glucose), or 150 h (SC + 4% glycerol).

To analyze how CoQ with long isoprenyl side chain affects the growth and viability of *S. cerevisiae*, we grew COQ1^SC^*,* COQ1^YL^, and WT on synthetic complete (SC) agar media containing glucose or glycerol as a non-fermentable carbon source (Fig. 6A). On the fermentable glucose, both strains grew like wild type, as expected. Only when grown on glycerol required active respiration, cells producing exclusively CoQ9 exhibited slower growth rates, compared to WT and COQ1^SC^. The respiratory defect in the CoQ9 was even more pronounced in liquid media when COQ1^YL^ cells went through a lag phase for several days (Fig. 6B). Notably, cells with an in-locus replacement of their *COQ1* gene also displayed delayed growth in liquid, indicating that not only the chain length but also the total levels CoQ were lower than those of the wild type and limited respiratory activity. Altogether, these results suggest that the longer CoQ9 can at least partially complement the native CoQ6 in *S. cerevisiae* and that the respiration of *S. cerevisiae* does not depend on the ubiquinone chain length.

### Prey discrimination of the *Saccharomyces* clade depends solely on the presence of CoQ6

**Fig 7:**
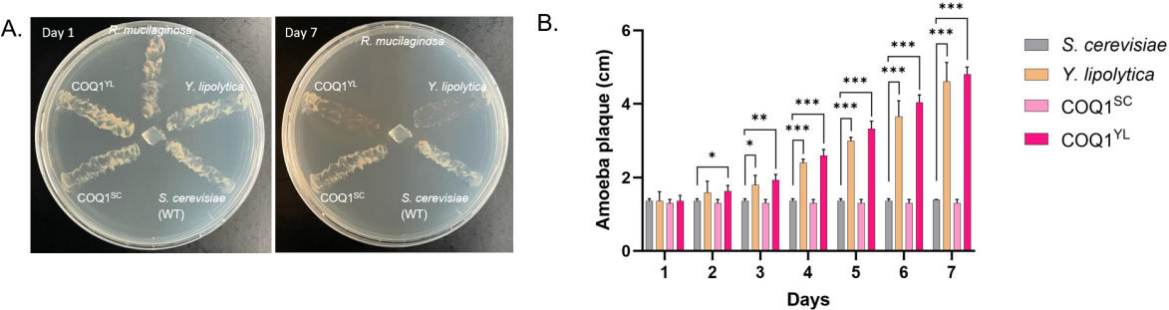
Growth of *P. aurantium* on different yeast strains. (**A**) *P. aurantium* was inoculated at the center of the plate (day 1) with streaks of *R. mucilaginosa*, *Y. lipolytica*, *S. cerevisiae* wild type, COQ1^SC^, and COQ1^YL^ on potassium phosphate agar plates. Plates were documented after 7 days of incubation, and feeding of the amoebae can be seen by the disappearance of the fungal cell streaks. (**B**) *P. aurantium* plaque formed by the feeding of *Y. lipolytica*, *S. cerevisiae* wild type, COQ1^SC^, and COQ1^YL^ strains on potassium phosphate agar plates was measured daily for 7 days and plotted on the graph. The data are represented as the mean values with error bars from three biological replicates. Significance levels are denoted with asterisks, where *P < 0.1; **P < 0.05; ***P < 0.01, based on *t*-test analysis.

As we expected that the *in vivo* exchange of CoQ6 by CoQ9 would alter prey discrimination by the amoeba, strains producing either CoQ6 (WT and COQ1^SC^) or CoQ9 (COQ1^YL^) were confronted with the fungivorous predator *P. aurantium*. Wild-type strains of the major food source *R. mucilaginosa*, a basidiomycete with CoQ10 (16), and wild-type cells of *Y. lipolytica* were included as further controls. On solid surfaces of agar plates, *P. aurantium* was unable to feed and proliferate on wild-type *S. cerevisiae* and COQ1^SC^ while cells of all other strains were readily consumed over a time course of 7 days (Fig. 7A). To quantify the growth of *P. aurantium*, plaque assays were performed. Moreover, the growth rate on *S. cerevisiae* cells producing CoQ9 was indistinguishable from the one when growing on *Y. lipolytica*, confirming that the lack of CoQ9 was the sole basis for prey discrimination of the *Saccharomyces* clade by the amoeba (Fig. 7B).

**Fig 8:**
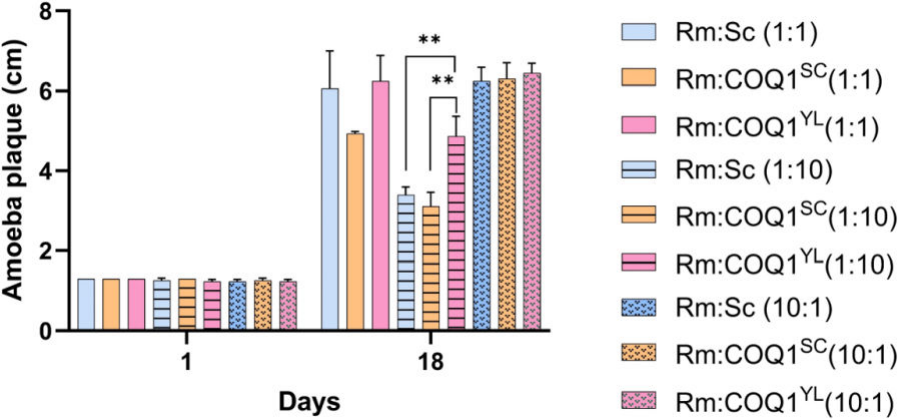
Growth of *P. aurantium* on a mixed diet of *R. mucilaginosa* and *S. cerevisiae* producing CoQ of different chain lengths. *P. aurantium* plaque formed by feeding different yeast combinations on potassium phosphate agarose plates was measured on day 1 and 18 and plotted on the graph. The data are represented as the mean values with error bars from three biological replicates. Significance levels are denoted with asterisks, where **P < 0.05, based on *t*-test analysis. Rm, *R. mucilaginosa*, Sc, *S. cerevisiae* wild type.

Furthermore, to analyze whether the presence of CoQ6 in *S. cerevisiae* (WT and COQ1^SC^) also helps it to evade better even in the presence of the food source *R. mucilaginosa*, we mixed *R. mucilaginosa* cells with *S. cerevisiae* (WT, COQ1^SC^, or COQ1^YL^) at different ratios (1:1, 1:10, or 10:1) and allowed *P. aurantium* to feed on them. As can be visualized (Fig. 8), *P. aurantium* displayed indistinguishable growth rates on WT, COQ1^SC^, or COQ1^YL^ when combined with *R. mucilaginosa* at 1:1 or 1:10 ratios. Nevertheless, *P. aurantium* displayed significantly higher growth rates for COQ1^YL^ compared to COQ1^SC^ when combined with *R. mucilaginosa* at a ratio of 10:1. Based on these results, we suggest that the presence of CoQ6 in the members of the *Saccharomyces* clade could act as a nutritional escape strategy in the micro-environment.

## DISCUSSION

In the natural habitat, fungi are attacked by many micro-predators. These include bacteria, unicellular amoeba, and fungi itself (9, 17–23). By contrast, to survive these hostile micro-predators, fungal species utilize several defense strategies such as cell wall reinforcement, production of secondary metabolites, or morphological shifts (1, 24). With the results of the present study, we suggest that CoQ6-based nutritional resistance may also contribute to predator escape and may have contributed to the maintenance of CoQ6 in the *Saccharomyces* clade. Recently, it has been shown that *P. aurantium* feeds on a broad range of fungal species, including the members of the *Candida* clade. *P. aurantium* and other members of the fungivorous Protostelia are frequently isolated from (decaying) leaf surfaces of deciduous trees (25), a habitat that is apparently shared with numerous species of yeast-like fungi, including *S. cerevisiae* and closely related species of its clade (26–28). Despite their likely co-occurrence and the ability to rapidly recognize and ingest cells of *Saccharomyces*, *P. aurantium* cannot maintain growth when these cells are the sole food source. However, *P. aurantium* could also metabolize non-food members of the *Saccharomyces* clade in the presence of a food source such as *R. mucilaginosa*, particularly when combined in a 1:1 ratio (4, 29). Furthermore, our experiments elucidate that *P. aurantium,* intriguingly, displays a remarkable 58% increase in growth when provided with a 10:1 ratio of COQ1^YL^ mixed with *R. mucilaginosa*, compared to COQ1^SC^, signifying the dynamic role of CoQ6 in predator-evasion.

From previous work, we could only identify two major molecular differences that clearly distinguish the two fungal clades and could thus be of relevance regarding the predatory profile of the amoebae. One is the alternative use of the CUG codon in the *Candida* clade. A group of budding yeast, including the human pathogen *C. albicans* and *C. parapsilosis,* has switched the translation of the codon CUG from normal Leucine to Serine (30, 31). However, this codon switch did not reveal any discernible difference in the feeding of *P. aurantium* and was thus excluded as a major predatory factor. The other notable difference is the unique use of CoQ6 as a short-chain ubiquinone in *S. cerevisiae* and clade members, whereas the *Candida* clade members possess CoQ9 as a major CoQ. The divergence in CoQ variants within the Saccharomycetaceae lineage, despite the ancestral presence of CoQ9 in Saccharomycetales, has remained enigmatic (7). In the late 1990s, Okada and colleagues showed that CoQ of varying isoprenyl chain length, even from bacteria, could complement the function of *COQ1* in *S. cerevisiae* (15). These results suggested a conserved role of CoQ in microbial physiology beyond species-specific CoQ variants. Ongoing research on the significance of diverse CoQ variants has unveiled new insights. A recent study highlights the crucial role of CoQ9 in conferring farnesol resistance in *C. parapsilosis* (8). These findings underscore the hidden multifaceted functions of CoQ within microorganisms, providing a broader understanding of its physiological impact beyond known functions in the cell.

Based on these findings and the fact that *Candida* clade members possess CoQ9 (6, 7), we investigated the previously annotated genome of *P. aurantium* for the presence of genes involved in coenzyme Q biosynthesis. Indeed, our results showed the presence of a *Protostelium* orthologue (PROFUN_07136) of *D. discoideum coq1*, responsible for CoQ10 biosynthesis. However, amino acid identity between the orthologs of the two amoebozoa members is only 26%, with the *Dictyostelium* Coq1 showing higher identity to orthologues from humans (41%) or yeast (46%). It may thus well be possible that none of the listed orthologues in Table 1 are functional in CoQ biosynthesis. From this perspective, it seems not surprising that many other CoQ biosynthesis genes, namely, *coq2*, *coq4*, *coq7*, *coq8*, and *coq9,* are absent in *P. aurantium*. Independent of the isoprenyl chain length *coq2*, a PHB-polyprenyl diphosphate transferase catalyzes the condensation of polyprenyl chain with PHB, *coq7*, catalyzes monooxygenation, and *coq8* acts as a protein kinase in CoQ biosynthesis. However, the functional characterization of *coq4* and *coq9* remained elusive (13). In accordance with the previous findings that the absence of *coq1-9* completely abolishes the coenzyme Q biosynthesis (13, 32, 33), we suggested the absence of functional coenzyme Q in *P. aurantium* originating from an intrinsic biosynthetic pathway.

As CoQ, an electron carrier in the electron transport chain, is indispensable for aerobic respiration in aerobic eukaryotes, such as *P. aurantium*, we concluded that any CoQ in the amoebae could be supplied from its fungal food source. Indeed, *in vitro* experiments revealed that, unlike coenzyme Q6, exogenous supplementation of 10 µM coenzyme CoQ9 or CoQ10 to the *S. cerevisiae* cells is sufficient to maintain the growth of *P. aurantium*. These results highlight the importance of functional CoQ biosynthetic machinery and align with the previous findings that 10 µM of exogenous coenzyme Q6 is required for the aerobic growth of *S. cerevisiae* with a defective CoQ biosynthesis pathway (34). These results have further supported our *in silico* findings and suggested that *P. aurantium* acquires coenzyme Q of a long polyprenyl side chain from its prey.

Studies with *S. cerevisiae* have shown that *coq* orthologs from many organisms, such as *Arabidopsis thaliana* and *Escherichia coli,* can complement the function of corresponding *coq* mutants in *S. cerevisiae* (35, 36). In line with these discoveries, our results showed that Yl-*coq1* complements the function of *COQ1* in COQ1^YL^ mutant and successfully produces CoQ9, albeit production levels likely remained lower than in the wild type. Especially when *S. cerevisiae* cells expressing the *Y. lipolytica* solanesyl polyprenyl synthase (COQ1^YL^) were forced to grow aerobically on a non-fermentable carbon source such as glycerol, growth decreased drastically. In previous studies, expression of different polyprenyl synthases imparted only minor or insignificant differences in the growth rate compared to the wild type, and the heterologous biosynthesis produced high levels of long-chain CoQ (8, 35). This apparent discrepancy could be explained by the fact that these *coq* genes were expressed from plasmids, and the study design was directed toward high expression and production levels. By contrast, our Yl-*coq1* and Sc*-coq1* were integrated into the chromosome of *S. cerevisiae* under the endogenous Sc-*COQ1* promotor.

As phylogenetically older fungal lineages represented by *Y. lipolytica* or *Schizosaccharomyces pombe* use the longer CoQ9 or CoQ10, the switch to CoQ6 by the *Saccharomyces* clade may have hampered respiration but became the sole factor that differentiates between prey and non-prey to a ubiquitous micropredator. It is thus tempting to speculate that the use of CoQ6 in the last common ancestor of the *Saccharomyces* clade served as a predatory escape factor and could possibly be maintained primarily in cells that thrive by fermentation.

## SUPPLEMENTAL MATERIAL

Supplemental data mbio.00342-24-s0001.pdf Supplemental figures and tables.

ASM does not own the copyrights to Supplemental Material that may be linked to, or accessed through, an article. The authors have granted ASM a non-exclusive, world-wide license to publish the Supplemental Material files. Please contact the corresponding author directly for reuse.

## References

[1] Radosa S, Saeed N, Hillmann F. 2023. Fungi and their environmental micropredators, p 207–225. In Pöggeler S, James T (ed), Evolution of fungi and fungal-like organisms. Springer International Publishing, Cham.

[2] Castellani A. 1930. An amoeba found in culture of yeast: preliminary note. J Trop Med Hyg 33:160.

[3] Hess S, Suthaus A. 2022. The vampyrellid amoebae (Vampyrellida, Rhizaria). Protist 173:125854. doi:10.1016/j.protis.2021.125854 35091168

[4] Radosa S, Ferling I, Sprague JL, Westermann M, Hillmann F. 2019. The different morphologies of yeast and filamentous fungi trigger distinct killing and feeding mechanisms in a fungivorous amoeba. Environ Microbiol 21:1809–1820. doi:10.1111/1462-2920.14588 30868709

[5] Kuraishi H, Itoh M, Katayama Y, Ito T, Hasegawa A, Sugiyama J. 2000. Ubiquinone systems in fungi. V. distribution and taxonomic implications of ubiquinones in Eurotiales, Onygenales and the related plectomycete genera, except for Aspergillus, Paecilomyces, Penicillium, and their related teleomorphs. Antonie Van Leeuwenhoek 77:179–186. doi:10.1023/a:1002416431944 10768477

[6] Suzuki M, Nakase T. 2002. A Phylogenetic study of ubiquinone-7 species of the genus Candida based on 18S ribosomal DNA sequence divergence. J Gen Appl Microbiol 48:55–65. doi:10.2323/jgam.48.55 12469316

[7] Diezmann S, Cox CJ, Schönian G, Vilgalys RJ, Mitchell TG. 2004. Phylogeny and evolution of medical species of Candida and related taxa: a multigenic analysis. J Clin Microbiol 42:5624–5635. doi:10.1128/JCM.42.12.5624-5635.2004 15583292 PMC535224

[8] Pathirana RU, Boone C, Nickerson KW. 2020. Longer ubiquinone side chains contribute to enhanced farnesol resistance in yeasts. Microorganisms 8:1641. doi:10.3390/microorganisms8111641 33114039 PMC7690737

[9] Hillmann F, Forbes G, Novohradská S, Ferling I, Riege K, Groth M, Westermann M, Marz M, Spaller T, Winckler T, Schaap P, Glöckner G. 2018. Multiple roots of fruiting body formation in Amoebozoa. Genome Biol Evol 10:591–606. doi:10.1093/gbe/evy011 29378020 PMC5804921

[10] Okada K, Suzuki K, Kamiya Y, Zhu X, Fujisaki S, Nishimura Y, Nishino T, Nakagawad T, Kawamukai M, Matsuda H. 1996. Polyprenyl diphosphate synthase essentially defines the length of the side chain of ubiquinone. Biochimica et Biophysica Acta (BBA) - Lipids and Lipid Metabolism 1302:217–223. doi:10.1016/0005-2760(96)00064-1 8765142

[11] Pierrel F, Hamelin O, Douki T, Kieffer-Jaquinod S, Mühlenhoff U, Ozeir M, Lill R, Fontecave M. 2010. Involvement of mitochondrial ferredoxin and para-aminobenzoic acid in yeast coenzyme Q biosynthesis. Chem Biol 17:449–459. doi:10.1016/j.chembiol.2010.03.014 20534343

[12] Sleda MA, Li Z-H, Behera R, Baierna B, Li C, Jumpathong J, Malwal SR, Kawamukai M, Oldfield E, Moreno SNJ. 2022. The heptaprenyl diphosphate synthase (Coq1) is the target of a lipophilic bisphosphonate that protects mice against Toxoplasma gondii infection. mBio 13:e0196622. doi:10.1128/mbio.01966-22 36129297 PMC9600589

[13] Kawamukai M. 2016. Biosynthesis of coenzyme Q in eukaryotes. Biosci Biotechnol Biochem 80:23–33. doi:10.1080/09168451.2015.1065172 26183239

[14] Awad AM, Bradley MC, Fernández-del-Río L, Nag A, Tsui HS, Clarke CF. 2018. Coenzyme Q(10) deficiencies: pathways in yeast and humans. Essays Biochem 62:361–376. doi:10.1042/EBC20170106 29980630 PMC6056717

[15] Okada K, Kainou T, Matsuda H, Kawamukai M. 1998. Biological significance of the side chain length of Ubiquinone in Saccharomyces Cerevisiae. FEBS Letters 431:241–244. doi:10.1016/S0014-5793(98)00753-4 9708911

[16] Yamada Y, Kondô K. 1973. Coenzyme Q system in the classification of the yeast genera Rhodotorula and Cryptococcus, and the yeast-like genera Sporobolomyces and Rhodosporidium. J Gen Appl Microbiol 19:59–77. doi:10.2323/jgam.19.59

[17] Old KM, Darbyshire JF. 1978. Soil fungi as food for giant amoebae. Soil Biol Biochem 10:93–100. doi:10.1016/0038-0717(78)90077-9

[18] Chakraborty S, Old KM, Warcup JH. 1983. Amoebae from a take-all suppressive soil which feed on Gaeumannomyces graminis tritici and other soil fungi. Soil Biol Biochem 15:17–24. doi:10.1016/0038-0717(83)90113-X

[19] Boer W de, Folman LB, Summerbell RC, Boddy L. 2005. Living in a fungal world: impact of fungi on soil bacterial niche development. FEMS Microbiol Rev 29:795–811. doi:10.1016/j.femsre.2004.11.005 16102603

[20] Karlsson M, Atanasova L, Jensen DF, Zeilinger S. 2017. Necrotrophic mycoparasites and their genomes. Microbiol Spectr 5:10.1128. doi:10.1128/microbiolspec.FUNK-0016-2016 PMC11687461 28281442

[21] Albuquerque P, Nicola AM, Magnabosco DAG, Derengowski L da S, Crisóstomo LS, Xavier LCG, Frazão S de O, Guilhelmelli F, de Oliveira MA, Dias J do N, Hurtado FA, Teixeira M de M, Guimarães AJ, Paes HC, Bagagli E, Felipe MSS, Casadevall A, Silva-Pereira I. 2019. A hidden battle in the dirt: soil amoebae interactions with Paracoccidioides spp. PLoS Negl Trop Dis 13:e0007742. doi:10.1371/journal.pntd.0007742 31589617 PMC6797224

[22] Németh MZ, Pintye A, Horváth ÁN, Vági P, Kovács GM, Gorfer M, Kiss L. 2019. Green fluorescent protein transformation sheds more light on a widespread mycoparasitic interaction. Phytopathology 109:1404–1416. doi:10.1094/PHYTO-01-19-0013-R 30900938

[23] Leveau JHJ, Preston GM. 2008. Bacterial mycophagy: definition and diagnosis of a unique bacterial–fungal interaction. New Phytol 177:859–876. doi:10.1111/j.1469-8137.2007.02325.x 18086226

[24] Künzler M. 2018. How fungi defend themselves against microbial competitors and animal predators. PLoS Pathog 14:e1007184. doi:10.1371/journal.ppat.1007184 30188951 PMC6126850

[25] Spiegel FW, Shadwick LL, Ndiritu GG, Brown MW, Aguilar M, Shadwick JD. 2017. Protosteloid amoebae (Protosteliida, Protosporangiida, Cavosteliida, Schizoplasmodiida, Fractoviteliida, and sporocarpic members of Vannellida, Centramoebida, and Pellitida), p 10–1007. In Handbook of the protists. Vol. 38. Springer, Cham.

[26] Sláviková E, Vadkertiová R, Vránová D. 2007. Yeasts colonizing the leaf surfaces. J Basic Microbiol 47:344–350. doi:10.1002/jobm.200710310 17645279

[27] Kowallik V, Greig D. 2016. A systematic forest survey showing an association of Saccharomyces paradoxus with oak leaf litter. Environ Microbiol Rep 8:833–841. doi:10.1111/1758-2229.12446 27481438

[28] Xia W, Nielly-Thibault L, Charron G, Landry CR, Kasimer D, Anderson JB, Kohn LM. 2017. Population genomics reveals structure at the individual, host-tree scale and persistence of genotypic variants of the undomesticated yeast Saccharomyces paradoxus in a natural woodland. Mol Ecol 26:995–1007. doi:10.1111/mec.13954 27988980

[29] Radosa S, Sprague JL, Lau SH, Tóth R, Linde J, Krüger T, Sprenger M, Kasper L, Westermann M, Kniemeyer O, Hube B, Brakhage AA, Gácser A, Hillmann F. 2021. The fungivorous amoeba Protostelium aurantium targets redox homeostasis and cell wall integrity during intracellular killing of Candida parapsilosis. Cell Microbiol 23:e13389. doi:10.1111/cmi.13389 34460149

[30] Santos MA, Tuite MF. 1995. The CUG codon is decoded in vivo as serine and not leucine in Candida albicans. Nucleic Acids Res 23:1481–1486. doi:10.1093/nar/23.9.1481 7784200 PMC306886

[31] Riley R, Haridas S, Wolfe KH, Lopes MR, Hittinger CT, Göker M, Salamov AA, Wisecaver JH, Long TM, Calvey CH, et al.. 2016. Comparative genomics of biotechnologically important yeasts. Proc Natl Acad Sci USA 113:9882–9887. doi:10.1073/pnas.1603941113 27535936 PMC5024638

[32] Ashby MN, Kutsunai SY, Ackerman S, Tzagoloff A, Edwards PA. 1992. Coq2 is a candidate for the structural gene encoding para-hdroxybenzoate: polyprenyltransferase. J Biol Chem 267:4128–4136. doi:10.1016/S0021-9258(19)50638-X 1740455

[33] Gin P, Clarke CF. 2005. Genetic evidence for a multi-subunit complex in coenzyme Q biosynthesis in yeast and the role of the Coq1 hexaprenyl diphosphate synthase. J Biol Chem 280:2676–2681. doi:10.1074/jbc.M411527200 15548532

[34] James AM, Cochemé HM, Smith RAJ, Murphy MP. 2005. Interactions of mitochondria-targeted and untargeted ubiquinones with the mitochondrial respiratory chain and reactive oxygen species. Implications for the use of exogenous ubiquinones as therapies and experimental tools. J Biol Chem 280:21295–21312. doi:10.1074/jbc.M501527200 15788391

[35] Okada K, Kainou T, Matsuda H, Kawamukai M. 1998. Biological significance of the side chain length of ubiquinone in Saccharomyces cerevisiae. FEBS Lett 431:241–244. doi:10.1016/s0014-5793(98)00753-4 9708911

[36] Ducluzeau A-L, Wamboldt Y, Elowsky CG, Mackenzie SA, Schuurink RC, Basset GJC. 2012. Gene network reconstruction identifies the authentic trans-prenyl diphosphate synthase that makes the solanesyl moiety of ubiquinone-9 in Arabidopsis. Plant J 69:366–375. doi:10.1111/j.1365-313X.2011.04796.x 21950843

