# Supplementary material for: The isoprenyl chain length of coenzyme Q mediates the nutritional resistance of fungi to amoeba predation
Source: mBio. 2024 May 15;15(6):e00342-24. doi: 10.1128/mbio.00342-24 (PMC11237637; doi:10.1128/mbio.00342-24)
Supplement: Supplemental data — Supplemental figures and tables. [file mbio.00342-24-s0001.pdf]

Supplemental material

Strains and Plasmids:

Table S1: The strains and plasmids used in this study.

Strains	Genotype	References
**Yeast**		
Saccharomyces cerevisiae BY4741 (WT)	*MATa*, *his3Δ1*, *leu2Δ0*, *met15Δ0*, *ura3Δ0*	Euroscarf
Saccharomyces cerevisiae Sc*coq1*::Sc*coq1* (COQ1^{SC})	*MATa*, *his3Δ1*, *leu2Δ0*, *met15Δ0*, *ura3Δ0*, Sc*coq1*::Sc*coq1*, KanXM^{R}	This study
Saccharomyces cerevisiae Sc*coq1*::Yl*coq1* (COQ1^{YL})	*MATa*, *his3Δ1*, *leu2Δ0*, *met15Δ0*, *ura3Δ0*, Sc*coq1*::Yl*coq1*, KanXM^{R}	This study
Saccharomyces cerevisiae YKK6	URA3^{+} *COQ1*::*URA3*	(Okada, Suzuki et al. 1996)
Saccharomyces cerevisiae SP1 harboring pYE6 plasmid		(Okada, Suzuki et al. 1996)
Saccharomyces cerevisiae SP1 harboring pYD10 plasmid	Ap^{r} LEU1; 1.2-kb BamHI-HindIII fragment from pCD10 in YEp13 M4	(Okada, Suzuki et al. 1996)
Yarrowia lipolytica	Wildtype strain, SF002693	Jena microbial resource collection
Rhodotorula mucilaginosa	Wildtype strain, SF10056	Jena microbial resource collection
**Amoeba**		
Protostelium aurantium var. *fungivorum*	Wild type strain	Hillmann et al., 2018
**Bacteria**		
Escherichia coli Top10 cells	Host strain for high copy number plasmid	Lab collection
**Plasmids**		
pJet_Sc*coq1*	Amp^{R}, KanXM^{R}, Sc*coq1*	This study
pJet_Yl*coq1*	Amp^{R}, KanXM^{R}, Yl*coq1*	This study
pJet1.2_rec	Amp^{R}, KanXM^{R}	This study

Primers:

Table S2: Primers used in this study are listed in Table.

**Oligonucleotides**	**Sequence 5' -> 3'**
pJet_BB_fwd	GACAAAAAACTATAAGTAACC
pJet_BB-rev	CAATGTGTTCATCTCCTTCG
SC_coq1-1	CGAAGGAGATGAACACATTGGCCAAAAATATAGTTGCCC
SC_coq1-2	GGTTACTTATAGTTTTTTGTCTTACTTTCTTCTTGTTAG
YL_coq1-1	CGAAGGAGATGAACACATTGGCCAACAACATTGGTTCTCTG
YL_coq1-2	GGTTACTTATAGTTTTTTGTCTTACTTGCTTCGGTTCATC
pJet_rec_fwd	GGTCCCCATTGGTTTCCTCG
pJet_rec_rev	CAGTTTGCTAGAACTATCGC
Sc_5' Check	GGAAAACAGTTCCTACAAGC
Sc_3' Check	GAGAGGCTTCGAGAGAGGAGG

Figure S1

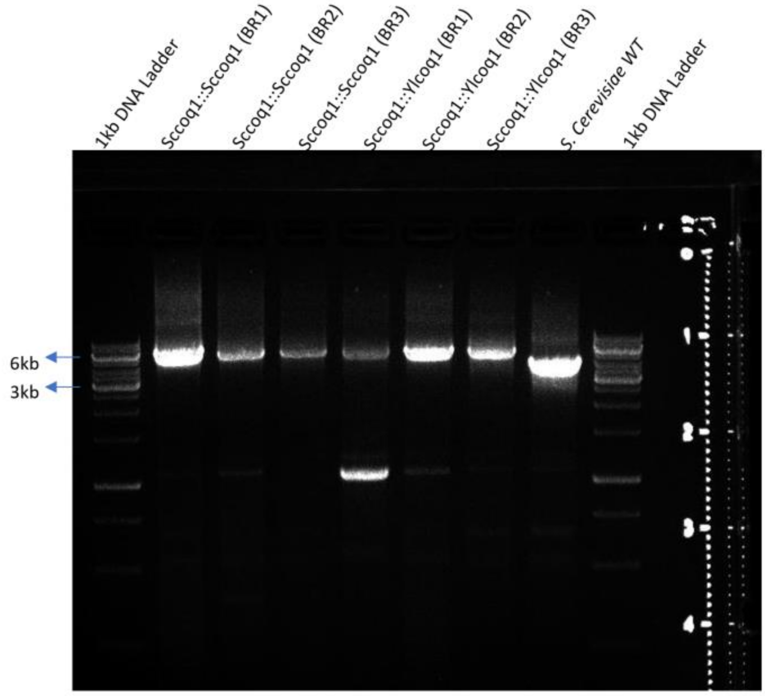

Figure S1: Agarose gel (1.5%) shows PCR products amplified with 5' check and 3' check primer pair. The homologous recombinants of *S. cerevisiae* containing *S. cerevisiae COQ1* ($COQ1^{SC}$) and *Y. lipolytica coq1* ($COQ1^{YL}$) exhibits an amplicon size of 5620 bp whereas an amplicon of 4302 bp can be visualized in *S. cerevisiae* WT strain.

Figure S2

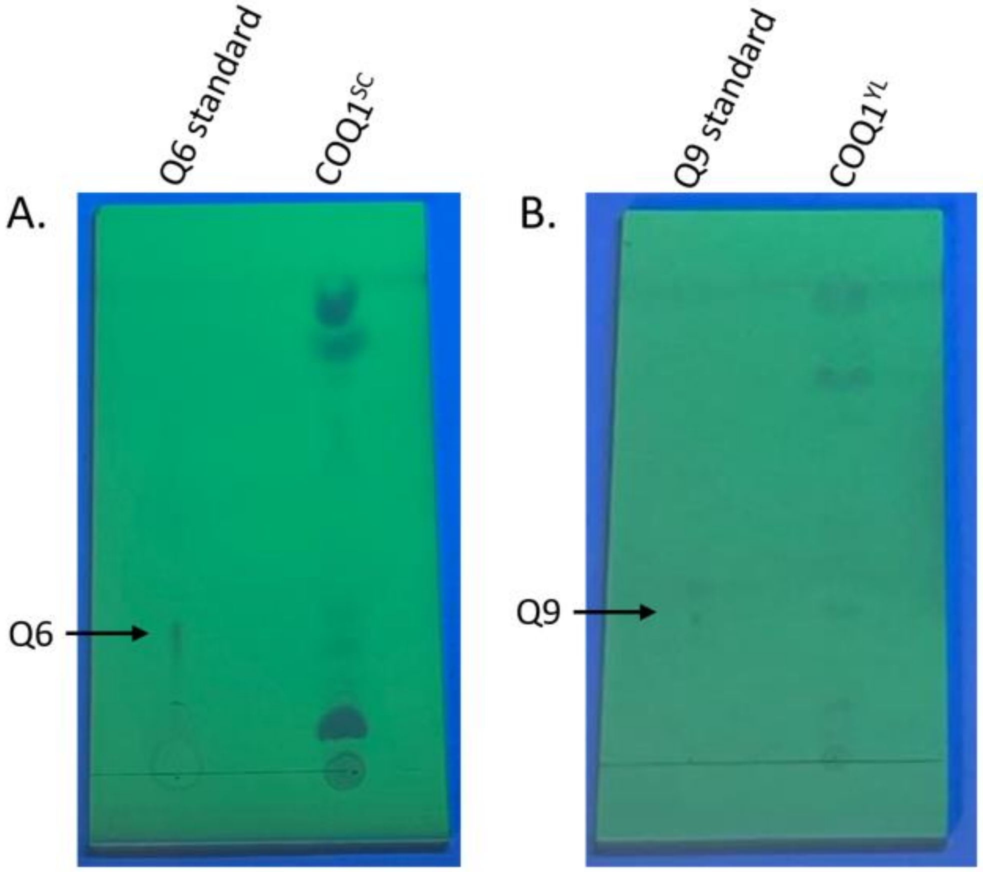

Figure S2: Analysis of the CoQs from *S. cerevisiae* ($COQ1^{SC}$ and $COQ1^{YL}$). A) CoQ6 is detected from $COQ1^{SC}$ on thin-layer chromatography. **B)** CoQ9 is detected from $COQ1^{YL}$ on thin-layer chromatography. Commercial standards of CoQ6 and CoQ9 were run in the same system.
